# Causes of hypercapnic respiratory failure: a population-based case-control study

**DOI:** 10.1186/s12890-023-02639-6

**Published:** 2023-09-14

**Authors:** Yewon Chung, Frances L. Garden, Guy B. Marks, Hima Vedam

**Affiliations:** 1 School of Clinical Medicine, South Western Sydney Clinical Campuses, Discipline of Medicine, UNSW Sydney, Sydney, Australia; 2 Department of Respiratory and Sleep Medicine, Liverpool Hospital, Locked Bag 7103 Liverpool, Liverpool, Sydney, NSW BC 1871 Australia; 3 Ingham Institute for Applied Medical Research, Liverpool, Sydney, Australia

**Keywords:** Hypercapnic respiratory failure, Hypercapnia causes, Hypercarbia causes, Ventilatory failure causes, Respiratory acidosis

## Abstract

**Objective:**

There are no population-based data on the relative importance of specific causes of hypercapnic respiratory failure (HRF). We sought to quantify the associations between hospitalisation with HRF and potential antecedent causes including chronic obstructive pulmonary disease (COPD), obstructive sleep apnea, and congestive cardiac failure. We used data on the prevalence of these conditions to estimate the population attributable fraction for each cause.

**Methods:**

A case–control study was conducted among residents aged ≥ 40 years from the Liverpool local government area in Sydney, Australia. Cases were identified from hospital records based on PaCO_2_ > 45 mmHg. Controls were randomly selected from the study population using a cluster sampling design. We collected self-reported data on medication use and performed spirometry, limited-channel sleep studies, venous sampling for N-terminal pro-brain natriuretic peptide (NT-proBNP) levels, and sniff nasal inspiratory pressure (SNIP) measurements. Logistic regression analyses were performed using directed acyclic graphs to identify covariates.

**Results:**

We recruited 42 cases and 105 controls. HRF was strongly associated with post-bronchodilator airflow obstruction, elevated NT-proBNP levels, reduced SNIP measurements and self-reported opioid medication use. There were no differences in the apnoea-hypopnea index or oxygen desaturation index between groups. COPD had the highest population attributable fraction (42%, 95% confidence interval 18% to 59%).

**Conclusions:**

COPD, congestive cardiac failure, and self-reported use of opioid medications, but not obstructive sleep apnea, are important causes of HRF among adults over 40 years old. No single cause accounts for the majority of cases based on the population attributable fraction.

**Supplementary Information:**

The online version contains supplementary material available at 10.1186/s12890-023-02639-6.

## Background

Hypercapnic respiratory failure (HRF) is a commonly encountered clinical scenario for hospital clinicians in a wide range of disciplines. Many patients are known to have a predisposing condition such as severe chronic obstructive pulmonary disease (COPD). However, in some patients presenting with HRF, the underlying cause is not apparent at initial presentation, and there may be multiple underlying potential causes existing concurrently. Although each cause requires disease-specific therapies, most patients require hospitalisation and many benefit from ventilatory support in dedicated respiratory and critical care units. Hence, HRF can be considered a single, albeit heterogeneous entity, that constitutes a significant problem for health facilities worldwide.

Previous studies examining the underlying causative conditions among patients with HRF have typically included participants identified following admission to an intensive care or respiratory admission and requiring ventilatory support therapy [1–5]. Three studies selected cases based on arterial blood gas (ABG) values [6–8]. and another relied on diagnosis codes suggestive for respiratory failure from hospital records [9]. Although these studies illustrate the range of conditions contributing to hospitalisation with HRF, none describe the prevalence of these factors in the source population and, hence, are unable to estimate the relative importance of each cause. Previous population-based studies have been limited to studying persons receiving home mechanical ventilation [10–12], which exclude cases whose underlying disease, comorbidities or socioeconomic factors preclude this intervention. Understanding the relative importance of the causes of HRF at a population level, irrespective of the treatments received, is required to develop investigation and management strategies for patients with undifferentiated HRF, and interventions to reduce hospitalisations associated with this condition.

Using a community-based case control study design, we sought to determine the strength of association between hospitalisation with HRF and the following conditions: COPD, congestive cardiac failure (CCF), obstructive sleep apnea (OSA), respiratory muscle weakness, and the use of opioid and benzodiazepine medications. We selected these causes in consensus based on previous studies and our clinical experience. In addition to estimating the strength of association with HRF, we used data on the prevalence of these conditions in the general community to estimate, for each cause, the population attributable fraction (PAF), an epidemiologic measure to describe the relative importance and public health impact of a risk factor in a population.

## Methods

### Overview

This prospective study was based in the City of Liverpool, a metropolitan area within Sydney, Australia [13]. The study population was restricted to persons aged 40 years and over. A case–control study design was implemented in which cases were people with HRF and controls were randomly selected members of the source population who did not have HRF. Non-English speaking persons were excluded if an interpreter was unavailable. We excluded nursing home residents due to difficulties in obtaining informed consent and accurate study measurements. Participants were reimbursed for their time with a gift card to the value of $40 AUD. Study procedures were approved by the South Western Sydney Local Health District Human Research Ethics Committee and all participants provided written, informed consent.

### Cases and controls

Cases were patients who attended Liverpool Hospital between 2016 and 2018. Potential cases were identified based on an ABG, collected within 24 h of presentation, demonstrating PaCO_2_ > 45 mmHg and pH ≤ 7.45. We anticipated the number of cases of HRF missed by this screening method to be low due to the increased risk of hospitalisation associated with hypercapnia, the public healthcare scheme in Australia that provides free hospital services to all citizens and most permanent residents, and local data that indicate most people with respiratory conditions from this population who require hospitalisation attend Liverpool Hospital. Medical records were reviewed to exclude suspected nosocomial cases of HRF, or instances where the person had suffered an out-of-hospital cardiac arrest or traumatic injury. Cases were invited to participate first by mail and then by follow-up telephone calls.

Population-based controls were randomly selected using a two-stage geography-based cluster sampling design, implemented from 2018 to 2019. First, we randomly selected 40 census tracts from the 449 comprising this region, the City of Liverpool. The probability of tract selection was proportional to the number of eligible residents in each tract. Next, we undertook ‘random walks’ to select households units within each tract, from which control participants were recruited. Investigators starting from the geographical centre of each tract walked along streets in directions guided by a computer-based random number generator. This method of population-based sampling is a practical method for random selection of participants from a large population when a population list is incomplete or unavailable, and is modified from methods used by the World Health Organization [14]. Letters of invitation were delivered to each household upon sampling, containing participant information sheets and multiple options for responding to the research team (telephone, e-mail, reply-paid envelope). All selected households received at least two letters of invitation and at least one home visit by study investigators in order to obtain a response, and record the number of eligible participants within each household.

### Procedures

Standardised questionnaires were administered by members of the research team. Data were collected on sociodemographic factors, comorbidities, and medications, including the use of opioids and benzodiazepines, in the preceding two years.

Spirometry was performed using the EasyOne spirometer (NDD Medical Technologies), before and after administration of salbutamol 200 μg via metered dose inhaler and spacer. All spirograms were reviewed by the author (Y.C.) for acceptability and repeatability using published criteria [15]. N-terminal pro-brain natriuretic peptide (NT-proBNP) levels were measured from venous blood samples by electrochemiluminescent immunoassay (Roche Diagnostics). Sniff nasal inspiratory pressure (SNIP) measurements were taken using a fitted nasal probe connected to a hand-held meter (MicroRPM, CareFusion). Up to 10 manoeuvres were performed per nostril [16]. Overnight home-based sleep testing was performed using a portable device with airflow (pressure cannula), respiratory movement and oximetry channels (ApneaLink, ResMed). All recordings were reviewed by the author (Y.C.) and excluded if there were fewer than 3 h of adequate flow and oximetry data. Data were scored automatically using ApneaLink software (V10.2). Apneas were defined as at least 90% decrease in airflow for at least 10 s and hypopneas as a decrease by 30% for the same duration associated with desaturation of 3% or more. Investigators conducting measurements were unblinded as to whether participants were cases or controls.

### Statistical analysis

Continuous variables are summarised as means with standard deviations (SD) and medians with interquartile ranges (IQR). Groups were compared using independent t-tests or Mann–Whitney U tests, as appropriate. Frequencies and percentages are used to describe categorical variables, and Fisher’s chi-square test used to compare groups. Baseline logistic regression models were used to assess the relationship between the presence or absence of HRF and continuous variables: the post-bronchodilator forced expiratory volume (FEV_1_)/forced vital capacity (FVC) ratio, NT-proBNP levels, maximum SNIP value (SNIP_max_) and apnea-hypopnea index (AHI). Receiver operator characteristic (ROC) curves were generated from these models to determine the area under the ROC curve (AUC) in order to assess the predictive value of each of these variables for HRF. For subsequent analyses, participants were classified as having COPD if post-bronchodilator FEV1/FVC was below the lower limit of normal, using Global Lung Initiative reference values [17]. The diagnosis of CCF was based on NT-proBNP levels ≥ 100 pmol/L (846 pg/mL). Respiratory muscle weakness was recorded if SNIP_max_ was less than 70 cmH_2_O and 60 cmH_2_O, among males and females, respectively. A diagnosis of moderate-to-severe OSA was recorded if the AHI was ≥ 15 events per hour.

**Fig. 1:**
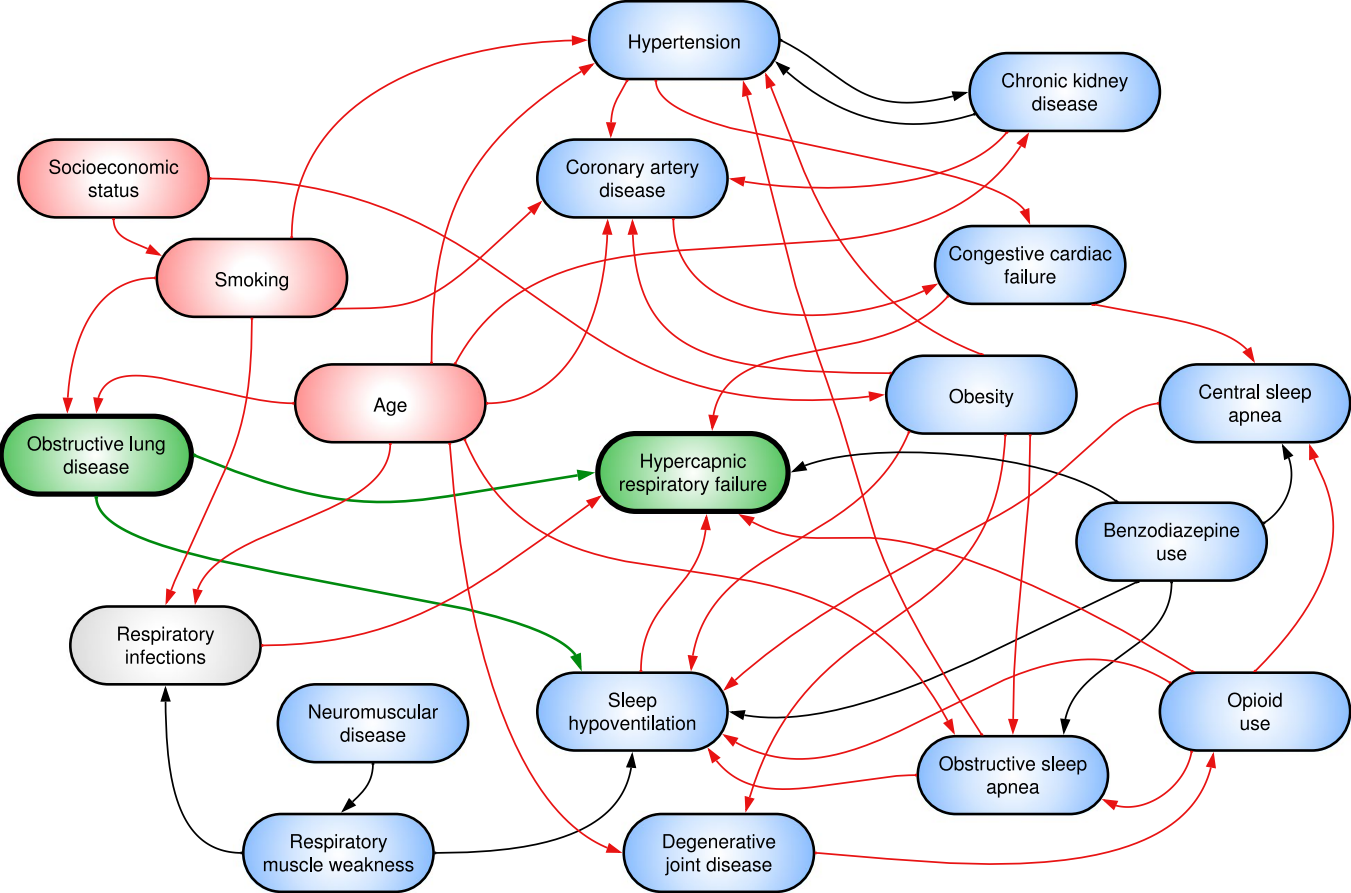
Causal diagram for hypercapnic respiratory failure. Directed acyclic graph illustrating assumed causal relationships between pre-specified exposure variables and the outcome of hypercapnic respiratory failure. In this case, obstructive lung disease has been selected as the exposure (green-shaded variable). Green arrows represent causal paths. Blue-shaded variables represent ancestors of the outcome and red-shaded variables represent ancestors of both the exposure and outcome. Red arrows represent biasing paths. Based on this diagram, the minimum adjustment set of variables to estimate the total effect of obstructive lung disease on hypercapnic respiratory failure are: age, smoking

We determined the adjusted association with HRF for each potential cause, reported as odds ratios (OR) with 95% confidence intervals (CI). Covariates for each model were informed by a directed acyclic graph (DAG) developed by the authors using the web-based program ‘daggity’ [18] showing direct and indirect pathways for the development of HRF (Fig. 1). Regressions were performed in SAS (Version 9.4). PAF estimates were calculated in STATA (Version 17). Full details of our regression models are provided in the Supplementary Material.

### Power calculation

We estimated 205 participants would be required in each group to detect an odds ratio of 2.8 and 2.0 for risk factors with prevalence values of 5% and 15%, respectively, with 80% power and two-sided alpha of 0.05. Study recruitment was stopped early due to low response rates and the COVID-19 pandemic.

## Results

**Fig. 2:**
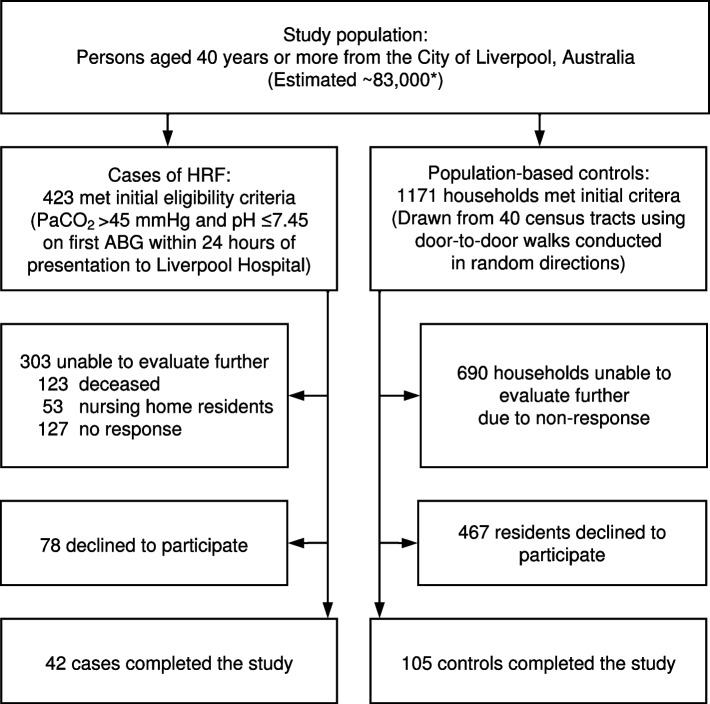
Participant flowchart. * Estimated source population is based on 2016 Census data (13)

One hundred and forty-seven subjects (42 cases and 105 controls) completed the study, as shown in Fig. 2.

Demographic and clinical characteristics are shown in Table 1. Compared with controls, cases were older, more likely to have been smokers, and had lower levels of educational attainment and household income. The most frequently reported respiratory conditions were asthma, COPD and OSA. Among cases, 34 (81%) reported at least one respiratory diagnosis, compared with 41 (39%) among controls (*p* < 0.001). The Charlson comorbidity index, a composite predictor of mortality [19], was significantly higher among cases (*p* < 0.001).

**Table 1:** Participant characteristics

	**Cases**	**Controls**	***P***
**Subjects** (n)	42	105	
**Age** (years, mean (SD))	66.8 (10.2)	59.4 (11.8)	< 0.001
**Sex**, Male (n (%))	21 (50%)	50 (48%)	0.9
**Body mass index** (kg/m^2^, mean (SD))	34.6 (11.0)	29.6 (6.1)	< 0.001
**Smoking exposure**			
Ever smoked (n (%))	36 (86%)	54 (52%)	< 0.001
Pack years (median (IQR))	45 (22.5 – 67.5)	22.5 (2.5 – 43.8)	0.04
**Highest level of educational attainment** (n (%))			0.02
Below upper secondary	23 (55%)	35 (33%)	
Upper secondary, post-secondary non-tertiary or tertiary	19 (45%)	70 (67%)	
**Household income range**^**a**^ (n (%))			< 0.001
Lowest quartile	1 (2%)	0 (0%)	
Medium lowest quartile	36 (86%)	54 (51%)	
Medium highest quartile	2 (5%)	23 (22%)	
Highest quartile	0 (0%)	22 (21%)	
No response	3 (7%)	6 (6%)	
**Self-reported past medical history** (n (%))			
Asthma	19 (45%)	26 (24%)	0.02
Chronic obstructive pulmonary disease	22 (52%)	16 (15%)	< 0.001
Pulmonary fibrosis	3 (7%)	1 (1%)	0.07
Bronchiectasis	1 (2%)	2 (2%)	1
Obstructive sleep apnea	18 (43%)	13 (12%)	< 0.001
Atrial fibrillation	11 (26%)	6 (6%)	0.001
Coronary artery disease	11 (26%)	8 (8%)	0.005
Congestive cardiac failure	14 (33%)	2 (2%)	< 0.001
Cerebrovascular accident	8 (19%)	4 (4%)	0.005
Peripheral vascular disease	4 (10%)	1 (1%)	0.06
Chronic kidney disease	7 (17%)	9 (9%)	0.2
Chronic liver disease	4 (10%)	1 (1%)	0.02
Hypertension	27 (64%)	37 (35%)	0.002
Diabetes mellitus	17 (40%)	18 (17%)	0.005
Solid organ malignancy	5 (12%)	6 (6%)	0.3
Depression, anxiety or other chronic mental health disorder	18 (43%)	20 (19%)	0.006
**Charlson Comorbidity Index** (median (IQR))	2 (1 – 5)	1 (0 – 1)	< 0.001

^a^Household income comparisons are based on 2016 Census data (13)

At least one pre-specified cause for HRF was identified in 42 (100%) cases, and 66 (63%) controls (*p* < 0.001) (Table 2). Among cases, 37 (88%) had two or more causes and 20 (48%) had three or more potential causes for HRF. The most common cause of HRF in cases was self-reported use of opioid medications (57%), followed by COPD (50%). Mean (SD) FEV_1_ in cases was 51 (21) percent predicted. The most frequent potential cause for HRF among controls was moderate-to-severe OSA (34%). The median (IQR) AHI among all controls was 6.6 (3.1, 19.1) events per hour.

**Table 2:** Prevalence of potential causes for hypercapnic respiratory failure among cases and control participants

**Cause**	**Cases *N* = 42**	**Controls *N* = 105**	***P***
*Chronic obstructive pulmonary disease*			
Frequency (FEV1/FVC < LLN, n/N (%))	21/42 (50%)	11/105 (11%)	< 0.001
Post-BD FEV_1_ (L, mean (SD))	1.27 (0.52)	2.44 (0.71)	
Post-BD FEV1 (% predicted, mean (SD))	51 (21)	90 (16)	
Post-BD FVC (L, mean (SD))	2.11 (0.59)	3.17 (0.88)	
Post-BD FVC (% predicted, mean (SD))	64 (15)	92 (13)	
FEV1/FVC < 0.7 (n/N (%))	26/42 (62%)	24/105 (23%)	
*Congestive cardiac failure*			
Frequency (NT-proBNP ≥ 100 pmol/L, n/N (%))	9/35 (26%)	1/81 (1%)	< 0.001
NT-pro-BNP (pmol/L, mean (SD))	101 (212)	14 (20)	0.003
*Obstructive sleep apnea*			
Frequency (AHI ≥ 15 events/hour, n/N (%))	7/25 (28%)	24/70 (34%)	0.6
AHI (mean (SD))	10.3 (8.6)	12.7 (13.2)	0.4
Oxygen desaturation index (mean (SD))	17.5 (11.2)	15.9 (14.2)	0.6
*Respiratory muscle weakness*			
Frequency (SNIP_max_ < LLN, n/N (%))	16/42 (38%)	11/103 (11%)	< 0.001
SNIP_max_ (cmH_2_O, mean (SD))	78 (24)	93 (28)	0.002
*Opioid use*			
Frequency (n (%))	24 (57%)	27 (26%)	< 0.001
*Benzodiazepine use*			
Frequency (n (%))	7 (17%)	14 (13%)	0.6

*AHI* Apnea-hypopnea index, *BD* bronchodilator, *FEV*_*1*_ Forced expiratory volume in the first second, *FVC* Forced vital capacity, *LLN* Lower limit of normal, *NT-proBNP* N-terminal brain natriuretic peptide, *SNIP*_*max*_ Maximum sniff nasal inspiratory pressure

The risk of HRF associated with each cause is shown in Table 3. CCF, COPD, respiratory muscle weakness and opioid use were strongly associated with the presence of HRF. The AUC values for FEV1/FVC, NT-proBNP, AHI and SNIP_max_ as continuous variables were 0.81, 0.74, 0.50 and 0.67, respectively. At the pre-specified cutoff, CCF had the strongest association (OR 13.4, 95% CI 1.40 – 128). Post-hoc analysis showed that when a lower NT-proBNP cut-off value of 35 pmol/L (296 pg/mL) was used to determine the presence of CCF, the magnitude of association was attenuated but remained statistically significant with an OR of 4.25 (95% CI 1.16 – 15.6).

**Table 3:** Risk of hypercapnic respiratory failure by potential underlying cause

	**Odds Ratio (95% CI)**		**Population attributable fraction**^**a**^** (95% CI)**
**Cause**	**Unadjusted**	**Adjusted**	
Chronic obstructive pulmonary disease	7.49 (3.16 – 17.8)	5.30 (1.95 – 14.4)	42% (18% – 59%)
Congestive cardiac failure	21.7 (2.80 – 166)	13.4 (1.40 – 128)	24% (7% – 38%)
Moderate-to-severe obstructive sleep apnea	1.14 (0.46 – 2.81)	0.38 (0.10 – 1.53)	*NA*
Respiratory muscle weakness	5.11 (2.11 – 12.3)	5.11 (2.11 – 12.3)	31% (11% – 46%)
Opioid use	3.85 (1.82 – 8.17)	3.64 (1.49 – 8.88)	41% (13% – 59%)
Benzodiazepine use	1.30 (0.48 – 3.49)	1.30 (0.48 – 3.49)	*NA*

^a^The population attributable fraction (PAF) was determined separately for each cause, and thus adds to more than 100%. The PAF was not determined when there was no significant relationship between the specified cause and hypercapnic respiratory failure

Moderate-to-severe OSA did not appear to be associated with increased risk of HRF, with more controls having this condition compared with cases. There was no difference between groups based on mean AHI, using a 4% desaturation threshold, or using the oxygen desaturation index. There were no significant differences in age, BMI, degree of comorbidity, reported sleepiness, and estimated risk of OSA based on the STOP-Bang score [20] between participants who did and did not complete the overnight sleep study.

No single cause was identified as being responsible for more than 50% of HRF cases, based on the PAF, as shown in Table 3. COPD had the highest adjusted PAF at 42% (95% CI 18% – 59%), followed by opioid use which had a PAF of 41% (13% – 59%). Despite its low prevalence in the general population (1%), CCF contributed substantially to the burden of HRF with a PAF of 24% (7% – 38%).

## Discussion

In this population-based case–control study, we show that HRF is a multifactorial condition with no single disease responsible for the majority of cases. In addition to chronic health conditions such as COPD and CCF, opioid use and respiratory muscle weakness are significantly associated with HRF hospitalisations. Interventions to reduce the prevalence of these causes have the potential to substantially reduce HRF-associated hospitalisations in this and other comparable populations.

Of the hypothesised causes, COPD had the highest population attributable fraction. Most previous surveys have shown COPD to be the dominant cause of hospitalisation with HRF [1–3, 7, 8], and COPD is also an important indication for treatment with home non-invasive ventilation therapy [12]. A prevalence study of hypercapnic COPD exacerbations has been used to approximate the requirements for hospital non-invasive ventilation services [21]. However, a considerable proportion of our cases did not have objective evidence of airflow obstruction, nor a history of chronic airways disease. Furthermore, we suspect the proportion of cases with COPD to be overrepresented in our study, as we have previously demonstrated that among cases of HRF, the presence of chronic airways disease is associated with a lower risk of death [22]. Hence, whilst COPD is an important contributor to HRF, it is not the only cause, and clinician judgment is required when assessing undifferentiated patients to identify alternative diagnoses.

In this population, CCF was an important contributor to hospitalisations with HRF. Patients with heart failure have reduced lung compliance and increased airways resistance, experiencing greater mechanical costs of breathing resulting in muscle fatigue and inability to maintain adequate ventilation [23]. Hypercapnia is present in up to 33% of patients with acute heart failure [24], and this can occur even in the absence of COPD [25]. One study of patients with compensated hypercapnia showed CCF to be more frequent than COPD [6]. The methods used to detect heart failure in previous studies have varied widely, ranging from clinician-reported diagnoses to echocardiographic findings [1, 3, 6]. We used the NT-pro-BNP to dichotomise patients, tolerating a degree of misclassification. NT-pro-BNP levels may be elevated with age, renal impairment and certain medications, but it has good negative predictive value when using low thresholds [26]. We selected a higher cut-off point to achieve greater specificity, and found the prevalence of CCF in controls to be comparable to previously published data [27]. Our study confirms the importance of CCF as a contributor to ventilatory failure, and the need to consider this cause among people presenting with HRF.

Self-reported use of opioid medications contributed significantly, with a PAF comparable to that of COPD. In recent decades, Australia and other high-income countries have documented a marked increase in the use of prescription opioids [28]. The clinical indication for most opioid prescriptions is acute pain [29], but chronic non-cancer pain is associated with continued opioid use [30]. Opioids directly suppress respiration but can also contribute to ventilatory failure indirectly via the mediators of sleep-related hypoventilation, obstructive and central sleep apnea [31]. Among adults with COPD, incident opioid prescriptions are associated with increased risk of adverse respiratory outcomes and death [32]. However, safety data among patients with chronic respiratory disease are inconsistent, particularly when considering those who receive opioids for refractory breathlessness [33]. Few previous studies have included drugs as a potential cause for HRF [3, 8], generally excluding such patients [1, 7]. We found opioid use to be associated with at least a three-fold increase in the risk of HRF, and hence may be an important modifiable risk factor in similar populations. Our results emphasise the need to rationalise opioid use to clinical situations where potential harms are outweighed by the benefits of these medicines.

There was a relatively high prevalence of respiratory muscle weakness among cases, and there was a significant association between reduced muscle strength and HRF. Neuromuscular disease including motor neuron disease, polio and muscular dystrophy accounts for a substantial proportion of home mechanical ventilation users [10]. We did not differentiate between such disorders and impaired respiratory muscle function due to other condition such as obesity and CCF, potentially leading to overestimates in PAF with respect to this risk factor.

Our study did not show a significant association between moderate-to-severe OSA and hospitalisation with HRF. Our analysis was based on the assumption that OSA could lead to HRF via sleep-related hypoventilation, or via an alternate pathway involving the development of CCF, with or without central sleep apnea and sleep hypoventilation [34] (Fig. 1). The apparent lack of association between OSA and HRF in our study might be explained by the very high prevalence of undiagnosed OSA among controls. A recent Australian study suggested the prevalence of moderate-to-severe OSA to be 20.2% in males and 10.0% of females [35]. However, a Swiss study demonstrated higher prevalence figures of 49.7% and 23.4% in males and females, respectively [36]. As such, whilst OSA is frequently diagnosed among patients with HRF, it might represent incorrect attribution as the cause of ventilatory failure. Alternatively, we may have missed a significant association due to the limitations of our testing methods. Previous studies have shown a high frequency of OSA among survivors of HRF, ranging from 51 to 83% when based on objective testing [1, 37]. The device we used in our study has 82% sensitivity for the diagnosis of moderate-to-severe OSA [38], but validation studies have typically excluded highly comorbid people and those with suspected hypoventilation. We also did not manually score data, relying on proprietary software for automatic detection of respiratory events. Hence, although we found a substantial prevalence of undiagnosed OSA which may be amenable to treatment, we did not find an association with HRF hospitalisations.

This study has some weaknesses. We were unable to achieve the recruitment target, but in our analysis we found that our sample size was sufficient to detect with statistical confidence the high magnitude of associations observed between HRF and the causes COPD, CCF and opioid use. Rather than the small groups per se, the primary limitation of this study is the potential selection bias induced by low response and non-participation rates. A relatively small proportion of potentially eligible people proceeded to study participation, despite attempts made by the investigators to minimise inconveniences and provide financial reimbursement for the time provided. Controls may have been more likely to participate if they had symptoms of, or were concerned about, OSA. If this effect had occurred in controls but not cases, it would have led to an underestimation of the association with OSA. Potential cases may have been excluded due to frailty or death. The effect of this selection bias would have been under-estimation of the association with causes known to increase the risk of death, such as CCF. Due to the case–control design, risk factors for the outcome are measured after the occurrence of HRF, although we expect most to be chronic conditions that would have been present prior to hospitalisation. Finally, our results may not be generalisable to other populations depending on socioeconomic and other factors affecting the prevalence of each cause.

Nevertheless, our work has several strengths distinguishing it from previous studies. This is one of few studies that have selected cases based on ABG results. We have provided objective measurements of causes rather than self-reported diagnoses or medical records which may be incomplete. We employed causal diagrams to inform our statistical analysis in keeping with modern epidemiological theory. Importantly, this is the first study of HRF to provide data on a control group, allowing estimation of the association between specific causes and HRF at a population level as well as the relative importance of each cause as reflected in the population attributable fraction.

## Conclusions

In summary, our study provides evidence for the multifactorial nature of HRF and the range of potential contributing factors. In addition to COPD, other causes including CCF, opioid use and respiratory muscle weaknesses are significantly associated with HRF hospitalisations. These findings have important implications for the assessment and management of patients with HRF and highlight the need for comprehensive evaluation to ensure that all treatable factors are addressed.

### Supplementary Information

**Additional file 1: Table E1.** Details of regression models used to determine the associations between each cause and the outcome of hypercapnic respiratory failure.

## Data Availability

The data that support the findings of this study are available on request from the corresponding author. The data are not publicly available due to privacy or ethical restrictions.

## Abbreviations

ABG: Arterial blood gas
AUC: Area under the ROC curve
BMI: Body mass index
CCF: Congestive cardiac failure
CI: Confidence intervals
COPD: Chronic obstructive pulmonary disease
DAG: Directed acyclic graph
FEV_1_: Forced expiratory volume in the first second
FVC: Forced vital capacity
HRF: Hypercapnic respiratory failure
IQR: Interquartile range
NT-proBNP: N-terminal pro-brain natriuretic peptide
OR: Odds ratio
OSA: Obstructive sleep apnea
PaCO_2_: Partial pressure of carbon dioxide in arterial blood
PAF: Population attributable fraction
ROC: Receiver operator characteristic
SD: Standard deviation
SNIP: Sniff nasal inspiratory pressure
SNIP_max_: Maximum sniff nasal inspiratory pressure
