# Supplementary material for: Causes of hypercapnic respiratory failure: a population-based case-control study
Source: BMC Pulm Med. 2023 Sep 14;23:347. doi: 10.1186/s12890-023-02639-6 (PMC10503117; doi:10.1186/s12890-023-02639-6)
Supplement: Supplementary file 1 — Additional file 1: Table E1. Details of regression models used to determine the associations between each cause and the outcome of hypercapnic respiratory failure. [file 12890_2023_2639_MOESM1_ESM.docx]

**Online Data Supplement**

Causes of hypercapnic respiratory failure:

a population-based case-control study

Yewon Chung, Frances L. Garden, Guy B. Marks, Hima Vedam

**Table E1. Details of regression models used to determine the associations between each cause and the outcome of hypercapnic respiratory failure**

| **Cause** | **Covariates** | **Auxiliary variables** |
| --- | --- | --- |
| Obstructive lung disease | - Age - Smoking (pack-years) | - Self-reported chronic obstructive pulmonary disease, emphysema OR chronic bronchitis |
| Congestive cardiac failure | - Coronary artery disease - Hypertension | - Self-reported congestive cardiac failure, atrial fibrillation, myocardial infarction OR coronary artery disease |
| Obstructive sleep apnoea | - Age - Obesity - Opioid use - Benzodiazepine use | - STOP-BANG score total |
| Respiratory muscle weakness | No adjustment required | - Age |
| Opioid use | - Age - Obesity | No missing data |
| Benzodiazepine use | No adjustment required | No missing data |

We estimated the crude and adjusted associations with hypercapnic respiratory failure (HRF) by fitting separate logistic regression models for each cause or exposure. The covariates used for each adjusted model were selected using a directed acyclic graph showing direct and indirect pathways for the development of HRF. If data were missing (e.g. if spirometry was unable to be performed to determine the presence or absence of obstructive lung disease), then auxiliary values were used to impute missing values using multivariate normal regression, using the *PROC MI* statement in SAS. Up to 40 imputed datasets were generated, depending on the fraction of missing information (FMI) for each model. Auxiliary values were identified by measuring their correlation (either alone or as a composite variable) with the exposure variable, and selecting those with Pearson correlation coefficient values of at least 0.4.
